# Exploring the connection between ego-resiliency and health behaviors: a cross-sectional study of Polish health sciences students

**DOI:** 10.1186/s12888-024-05617-2

**Published:** 2024-02-27

**Authors:** Małgorzata Dębska-Janus, Paweł Dębski, Agnieszka Nawrocka, Jacek Polechoński, Wojciech Madejczyk, Karina Badura-Brzoza

**Affiliations:** 1 Institute of Sport Sciences, Academy of Physical Education in Katowice, Mikołowska 72A, 40-065 Katowice, Poland; 2 Department of Psychiatry, Faculty of Medical Sciences in Zabrze, Medical University of Silesia in Katowice, Pyskowicka 49, 42-612 Tarnowskie Góry, Poland

**Keywords:** Health behaviors, Ego-resiliency, Optimal regulation, Openness to life experiences, Students

## Abstract

**Background:**

The aim of this study was to examine the relationship between ego-resiliency and the intensity of health behaviors among Polish health sciences students.

**Methods:**

The study involved 483 students from health-related faculties in southern Poland, consisting of 314 women (63.7%) and 179 men (36.3%). The average age of the participants was 21.7 ± 2.5 years. To assess resiliency (ER), the Ego-Resiliency Scale (ER89-R12) by Block and Kremen was used in its Polish adaptation. The intensity of health behaviors was examined using the Health Behavior Inventory (HBI) developed by Z. Juczyński.

**Results:**

The results revealed a positive correlation between the intensity of health behaviors and ER (*r* = 0.43, *p* < 0.001), both for the general factor and its categories (positive mental attitude, proper eating habits, preventive actions, and prohealth activities). Students with a high level of health behaviors exhibited significantly higher ER (*M* = 38.95, SD = 5.15) compared to those with average (*M* = 35.93, SD = 5.03) and low (*M* = 32.97, SD = 5.12) HBI levels. Among the HBI categories, Positive Mental Attitude showed the strongest correlation with both general ER and its factors: optimal regulation (OR) and openness to life experiences (OL). Furthermore, the correlation was found to be stronger with the OR and weaker with OL.

**Conclusion:**

Higher ER in students is correlated with a greater frequency of health behaviors. Nurturing the development of ER may contribute to the maintenance of prohealth practices despite life difficulties and temporary loss of motivation. This, in turn, promotes the regularity of health behaviors, which is crucial for their positive impact on overall health.

## Background

According to the concept of salutogenesis, health is a continuous process of seeking and maintaining balance amidst the constant burdens and demands of life [1]. The foundation for maintaining homeostasis is the ability to adapt to environmental, mental, and social stimuli in a manner that does not disrupt the structure and functioning of the individual in their biopsychosocial dimension [2]. The conservation of resources theory proposed by Hobfoll [3] states that the effectiveness of this process is influenced, among other factors, by the resources available to individuals, which impact their motivation to protect and enhance their health, limit activities that pose health risks, and facilitate the recovery process in case of health deterioration [3]. These adaptive potentials encompass personal and social factors, as well as attributes of the physical and natural environment that alleviate life stressors [4]. Five psychological resources have been identified as crucial for protecting mental health: a sense of control, self-efficacy, coherence, resiliency, and social support [3].

Resiliency can be understood both as a personality trait that contributes to the consistent prohealth regulation of behavior and as a dynamic adaptive process that arises in response to crises (resilience). As a personality trait, resiliency determines an individual’s capacity to adapt flexibly and effectively to both external and internal stressors [5]. It plays a crucial role in guiding the adaptive regulation process to navigate changing circumstances and difficulties, facilitating healthy functioning even in unfavorable conditions [6]. Due to its strong association with flexible self-regulation, this trait is commonly referred to as ego-resiliency (ER) in the literature [5]. ER is a relatively recent concept in research theory and practice, primarily explored by health and stress psychologists. Investigations into resilience as an adaptive process and ER as a personality trait have shed light on the diverse functioning of individuals facing crises and traumatic situations. Several researchers have recognized the multifaceted nature of ER as a trait [6–9]. It has been found to correlate with positive emotions such as optimism, self-efficacy, strong social relationships, and positive self-esteem [4, 8]. These factors may account for its ability to rebound from negative experiences in difficult situations (referred to as bounce-back) and maintain normal activities and a general openness to new experiences [10]. On the other hand, Block and Kremen emphasized the link between ER and self-control, describing it as a quality that enables adaptive flexibility in adjusting the level of control based on the situation at hand [6].

Previous research has consistently demonstrated the beneficial effects of ER on various aspects of functioning. ER has been found to facilitate adaptation to the requirements of subsequent stages of life [11, 12], and positively correlates with the quality of life in both healthy and clinical populations [13, 14], influence the speed of recovery and return to normal functioning following traumatic experiences such as armed conflicts, natural disasters, epidemics, and social pathologies [15, 16], and impact emotions [17–21].

Furthermore, a few studies have highlighted a positive relationship between mental resilience and engagement in habitual physical activity [22, 23], a greater tendency to adopt principles of rational nutrition [24], and the avoidance of risky behaviors [21, 25]. With its association with adaptive self-control, optimism, and self-efficacy, ER may facilitate the adoption of health-oriented lifestyle changes. It is particularly useful in maintaining consistent engagement in prohealth practices despite life challenges and temporary loss of motivation. This consistency is crucial for maximizing the positive impact of health behaviors on overall well-being [23, 26].

Simultaneously, adopting a healthy lifestyle plays a crucial role in the primary and secondary prevention of chronic diseases, which can lead to a decrease in quality of life, disability, and premature mortality. Understanding the resources that contribute to individuals’ inclination to engage in regular health behaviors, particularly those that can be modified, is vital for developing effective health promotion programs and interventions. Therefore, exploring the relationship between ER and the intensity of health behaviors among individuals from diverse social groups becomes an intriguing research topic within the field of public health. To date, this relationship has been examined in Korean children [27] and Polish seniors [28].

In view of the above, the aim of our study is to investigate the correlation between ER and the extent of health behaviors in Polish students. Combining the existing knowledge we assume that there may be a positive relation between the intensity of ego-resiliency and the frequency of health behaviors in this group of people. It is the first research exploring this relation it in the group of students.

## Methods

The study employed a cross-sectional design and included health sciences students from the Silesian Medical University and Academy of Physical Education in Katowice, Poland. The purposive sampling was used to check the relation in a group of people who tend to be aware of the role of health behaviours and therefore may have the positive cognitive component of attitude towards them and be more likely to execute them. All students from the two faculties offering health-related programs (Medical and Physical Education Faculties, *N* = 1500 students) were invited to participate in the research by electronic platform used to communicate with students (MS Teams) The information sessions with potential participants (800 students) were organized in direct contact and via electronic platform. They were presented with the study’s objectives, protocol, and research instruments. A power analysis approach was used for the sample size calculation. The inclusion criteria for participation in the study were providing consent to participate and completing the entire study program. Participants who reported receiving treatment for any mental disorders within the previous year and those with missing data were excluded from the final analysis.

Considering the primary aim of our study we set the following parameters for the analysis: an alpha level of 0.05, a power of 0.80 and an anticipated medium effect size based on Cohen’s guidelines.The estimated minimal sample size for the assumed criteria was 400 participants. To account for potential data loss and non-responses, we increased this estimate by approximately 20%, resulting in a target sample size of around 480 participants. Ultimately, data from 483 students with complete information were included in the analysis. The research group comprised 314 women (63.7%) and 179 men (36.3%). The gender distribution reflected the typical representation among students at the surveyed universities (Silesian Medical University: women: 68% men: 32%, Academy of Physical Activity: women: 58%, men: 42%). The average age of the participants was 21.7 ± 2.5 years (21.9 ± 2.8 for students from Silesian Medical University, 21.4 ± 2.0 for students from the Academy of Physical Education). The majority of the respondents, 78.9%, resided in urban areas, while the remaining 21.1% lived in rural areas.

Psychological tests were administered to collect the necessary research data. The level of ER was assessed using the Polish adaptation of Block’s and Kremen’s Ego-Resiliency Scale (ER89-R12) [6], which was developed by Kołodziej-Zaleska and Przybyła-Basista [29]. To measure the intensity of health behaviors, the Health Behavior Inventory (HBI) developed by Z. Juczyński [30] was utilized. The research took place in University laboratories during special meetings under the guidance of a psychologist in the second quarter of 2021.

### ER scale

The Polish version of the scale consists of 12 statements that assess two factors of ER: optimal regulation (OR) and openness to new life experiences (OL). The OR factor includes statements 6–9, while the OL factor comprises questions 1–5 and 10–12. Respondents rate their level of agreement with each statement on a four-point scale, with 1 indicating “I do not agree at all” and 4 indicating “I agree very strongly.” The overall ER score is calculated by summing the scores from all the statements, ranging from 12 to 48 points. A higher score reflects greater resilience in the individual being assessed. The OR and OL subscale scores are obtained by summing the scores assigned to the statements corresponding to each factor. The reliability of the scale is reported as 0.80 for the general scale, 0.77 for the OR subscale, and 0.59 for the OL subscale [29]. In the current study, Cronbach’s alpha coefficients were calculated as 0.76 for the entire scale, 0.75 for OR, and 0.60 for OL.

### Health behaviors inventory

The scale consists of 24 statements that assess health behaviors across four categories: positive mental attitude (coping with stress,emotional self-control, avoiding excess psychological tension) proper eating habits (dietary diversity, fiber intake, vegetable and fruit intake), preventive actions (compliance with medical recommendations, knowledge of emergency numbers, health literacy), and prohealth activities (undertaking physical activity, sleep hygiene, adequate physical regeneration, leisure activities). Respondents rate the frequency of engaging in each action on a five-point scale (1–5), where 1 represents “almost never,” 2 represents “rarely,” 3 represents “occasionally,” 4 represents “often,” and 5 represents “almost always.” The general HBI is obtained by summing the scores from all the questionnaire items. These raw scores are then transformed into Standard Ten Scores (1–10 Sten), where Sten scores of 1–4 represent low intensity, 5–6 signify average intensity, and 7–10 indicate high intensity. The specific intensities of health behaviors within each category are calculated by summing the scores of the relevant statements according to the questionnaire’s key [30]. The reliability of the tool, measured by Cronbach’s α coefficient, is reported as 0.85 for the entire inventory, while for the individual subscales, it ranges from 0.60 to 0.65 [30]. In the current study, Cronbach’s α coefficients are 0.85 for the entire scale and range from 0.68 to 0.80 for the subscales.

The research procedure was approved by ethical approval from the Research Ethics Committee of the Medical University of Silesia in Katowice (PCN/0022/KB/277/19). Before data collection, all participants were informed about the aim of the study. They voluntarily participated in the research and had the right to withdraw their participation at any time. Written consent was obtained from each participant for the use of the collected examination.

### Statistical analysis

Descriptive statistical parameters such as arithmetic means (M), standard deviations (SD), minimum (Min), and maximum (Max) values were computed. The reliability of the measures used was assessed by calculating Cronbach’s α coefficients in the study sample. Qualitative data were presented using the structure index. The Shapiro-Wilk’s tests were conducted to check the normality of distribution of quantitative variables. Their results confirmed the normal distribution for them. To examine the relationship between ER and health behaviors, Pearson’s correlation analysis was conducted. In addition, a one-way analysis of variance (ANOVA) for independent samples was performed, followed by multiple Bonferroni tests, to determine if there were significant differences in ER across different levels of health behavior intensity (low, average, and high).

## Results

The participants had an average score of 81.39 points for health behaviors (Table 1). The scores for individual categories of health behaviors were similar, ranging from 20.29 to 20.41 points. Participants scored highest in the preventive behaviors category, while the lowest scores were observed in the domain of health practices. By converting the scores to a sten scale, three levels of health behaviors were identified. Only 28% of the participants were classified as having a high level of health behaviors, while the majority of students (39.1%) were categorized as having an average level of health behaviors (Table 1).

**Table 1:** Basic characteristics of health behaviors

Variables		M	SD	Min	Max	N	%
Indicator of health behaviors (24–120)		81.39	13.66	34	120		
Positive mental attitude (6–30)		20.34	4.57	7	30		
Healthy eating habits (6–30)		20.35	5.02	7	30		
Preventive behaviors (6–30)		20.41	4.41	8	30		
Prohealth Activities (6–30)		20.29	4.12	9	30		
Level of health behaviors:							
	Low (1–4 Sten)					162	32.9%
	Average (5–6 Sten)					193	39.1%
	High (7–10 Sten)					138	28.0%

The participants exhibited an average level of ego resiliency at 35.8 points (Table 2). None of the respondents scored below 18 points, and approximately 48.7% of the participants achieved a total score exceeding 36. Regarding the specific aspects of ego resiliency, participants attained an average score of 23.53 points in the OR category and 12.27 points on the scale measuring openness to new life experiences.

**Table 2:** Basic characteristics of ego-resiliency

Variables	M	SD	Min	Max
Ego-resiliency (12–48)	35.80	5.59	18	48
Optimal regulation (0–32)	23.53	4.11	10	32
Openness to new life experiences (0–16)	12.27	2.51	4	16

A moderate positive correlation was identified between ego resiliency and the overall intensity of health behaviors (*r* = 0.43, *p* < 0.001) (Table 3). Significant positive correlations were observed between ego resiliency, its components, and all categories of health behaviors. The most robust positive relationship was observed between positive mental attitude and OR (*r* = 0.51, *p* < 0.001). The weakest correlation was found between openness to new life experiences and health practices (*r* = 0.13, *p* < 0.01).

**Table 3:** The relationship between mental resilience and health behaviors (Pearson correlation)

Variables	Ego-resiliency	Optimal regulation	Openness to new life experiences
Health behaviors	0.43^**^	0.42^**^	0.28^**^
Positive mental attitude	0.48^**^	0.51^**^	0.22^**^
Healthy eating habits	0.29^**^	0.25^**^	0.23^**^
Preventive behaviors	0.30^**^	0.25^**^	0.25^**^
Prohealth activities	0.23^**^	0.24^**^	0.13^*^

*p < 0.01, **p < 0.001

The results of the one-way ANOVA demonstrate that the overall ego resiliency score and its subscales are influenced by the level of health behaviors (Table 4). In the case of ego resiliency and OR, a strong effect was observed (*F* = 51.48, *p* = 0.000, *η*^2^ = 0.17; *F* = 51.21, *p* = 0.000, *η*^2^ = 0.17). Similarly, a significant relationship was found for openness to new life experiences, although the effect size was moderate (*F* = 15.96, *p* = 0.000, *η*^2^ = 0.06).

Additional analyses indicate significant differences among all compared groups. Individuals with high levels of health behaviors exhibited the highest ego resiliency (*M* = 38.95, SD = 5.15). The mean score in this group was significantly greater compared to individuals with average levels of health behaviors (*M* = 35.93, SD = 5.03) and those with low levels of health behaviors (*M* = 32.97, SD = 5.12).

**Table 4:** One-way analysis of variance (ANOVA)

Variables	Level of health behaviors						F (2.490)	p	η^2^	Bonferroni test
	Low		Average		High					
	M	SD	M	SD	M	SD				
Ego-resiliency	32.97	5.12	35.93	5.03	38.95	5.15	51.48	0.000	0.17	1 < 2 < 3
Optimal regulation	21.43	3.56	23.66	3.77	25.81	3.93	51.21	0.000	0.17	1 < 2 < 3
Openness to new life experiences	11.54	2.66	12.26	2.33	13.14	2.30	15.96	0.000	0.06	1 < 2 < 3

## Discussion

During the conducted research, it was discovered that mental resilience plays a significant role in the healthy psychological functioning of individuals [11–15, 18, 21, 31, 32]. This quality, which involves the ability to adapt self-control to situational demands and is positively correlated with optimism and self-efficacy, has been found to support the implementation of health-promoting lifestyle changes in various studies [21, 23, 25, 26, 33]. Therefore, the aim of this study was to examine the relationship between mental resilience and the intensity of health-related behaviors among Polish students.

Based on our research findings, the average level of psychological resilience among the participants was 36 out of a maximum score of 48. This average level is comparable to that of students in health sciences from South Korea, the United Kingdom, and Poland [34–37]. In terms of overall health behaviors, at least 67.1% of the participants in our study exhibited an average or higher level (≥ 5 Sten score). This result is slightly more favorable compared to findings from similar studies involving student populations [38, 39].

Our study revealed a moderate positive correlation between psychological resilience and the overall measure of health behaviors among the surveyed students. This relationship has previously been investigated among Polish seniors [28]. These publications have indicated that a higher level of ego resilience is associated with a health-promoting lifestyle. It is important to note that these studies employed different research instruments and did not specifically examine the relationship between ego resilience and its components with specific categories of health behaviors.

In relation to behaviors primarily focused on physical health, such as healthy eating habits and prohealth activities, positive correlations were also observed, although their strength was comparatively weaker. Other studies conducted by various authors have similarly confirmed the relationship between the intensity of specific behaviors related to physical health, including physical activity [22, 23], healthy eating habits [27, 40, 41, 42], and avoidance of risky behaviors [25], with the level of psychological resilience. This association can be explained by the enhanced capacity for adaptive self-regulation exhibited by individuals with higher levels of ER [5, 6]. Such resilience facilitates overcoming daily challenges in achieving health-related goals, including those related to a health-promoting lifestyle [22, 23, 43]. Consequently, individuals with high resilience are more likely to maintain consistency in their behaviors, which is a crucial factor in attaining the health benefits associated with health-related behaviors.

The correlation between the intensity of self-regulation and its components with the level of OR (*r* = 0.42, *p* < 0.001) was found to be stronger than in the case of openness to experience (*r* = 0.28, *p* < 0.001). This can be attributed to the robust association between resiliency and flexible self-regulation, which plays a crucial role in adapting to changing life circumstances and overcoming challenges [6]. In validation studies of the Polish version of the ER-89-R12 questionnaire used in this research, the OR subscale exhibited a stronger correlation with the overall ER score (0.920) compared to the openness to experience scale (0.787).

The findings of our study hold significant implications for public health interventions. Low adherence to health behaviors is a major contributor to chronic diseases and premature mortality. Ego resiliency emerges as a supportive factor in maintaining a healthy lifestyle, and evidence from several studies demonstrates the modifiability of this trait [44, 45]. Therefore, promoting the development of ego resiliency should be incorporated into modern, comprehensive preventive interventions.

Combining the existing knowledge on the impact of ego resiliency on health, its potential for modification and positive relation with health behaviors, health-related lifestyle changes may be supported by the enhancement of ego resiliency. Developing self-regulation in variuos life situations is connected with general cognitive performance and components like mindfulness, acceptance, insight into values, cognitive defusion, engagement and. The use of different techniques aimed at developing the above-mentioned cognitive components and functions, typical for cognitive-behavioral therapeutic strategies, may be useful. It seems to be reasoned to introduce psychoprophylaxis into the study programmes for health sciences students as a subject introducing this knowledge and skills. ER can be practiced in daily activities by changing conditions or introducing disturbing stimuli whilst performing everyday, well-known tasks, e.g. daily rituals, leisure-time habits. Regular tai-chi trainings or active relaxation in the nature are confirmed to be effective in ER improvement [44, 45].

It is important to note that this study serves as a preliminary report on the relationship between the overall intensity of health behaviors and ego resiliency in Polish students. Three limitations should be acknowledged. Firstly, the obtained results primarily reflect students who are engaged in health education programs, potentially indicating a higher level of health awareness compared to the general young adult population. Therefore, future studies should include students from different universities and disciplines unrelated to health sciences to ensure a more diverse sample. Secondly, in this study, the intensity of health behaviors was subjectively assessed using a questionnaire. Future research would benefit from verifying these behaviors more accurately by incorporating objective measures and reassessing their relationship with ego resiliency. Lastly, investigating the causality of this relationship is crucial. Further investigations should explore the causal nature of the relationship between ego resiliency and the intensity of health behaviors.

## Conclusion

There exists a significant and moderately strong positive correlation between the overall intensity of health behaviors and ego resiliency among students. This correlation is evident across all categories of health behaviors as well as in overall psychological resilience and its constituent factors. Notably, behaviors associated with mental health (positive mental attitude subscale) demonstrate the strongest correlation with ego resiliency. Furthermore, it has been observed that the intensity of health behaviors exhibits a stronger correlation with the OR than OL factor of ego resiliency. The level of resilience varies significantly depending on the degree of engagement in health behaviors, with higher levels of resilience observed among students with high levels of health behaviors and lower levels of resilience observed among those with low levels of such behaviors.

## Data Availability

The datasets used and/or analysed during the current study are available from the corresponding author on reasonable request.

## Abbreviations

ER: ego resiliency
HBI: Health Behavior Inventory
OR: optimal regulation
OL: openness to new life experiences
M: arithmetic means
SD: standard deviations
Min: minimum
and Max: maximum
N: number
